# Red Light Therapy Attenuates Prolonged LED light Exposure‐Associated Neuropathology and Mediates Circadian Clock Genes ‐ *Per*1 and *Bmal*1 Expression in Rats' Basal Ganglia

**DOI:** 10.1002/alz.084765

**Published:** 2025-01-03

**Authors:** Adejoke Olukayode Obajuluwa, Laoye John Babafemi, Ogochukwu Jessica Ndianefo, Tiwalola Madoc Obajuluwa, James Chrystopher Lech, Pius Abimbola Okiki

**Affiliations:** ^1^ Afe Babalola University, Ado Nigeria; ^2^ Afe Babalola University, Ado ‐Ekiti Nigeria; ^3^ Afe Babalola University, Ado‐Ekiti Nigeria; ^4^ University Amsterdam Medical Centre, Amsterdam Netherlands

## Abstract

**Background:**

Prolonged exposure to LED‐light has been associated with impaired sleep quality and pathogenesis of various diseases, including Alzheimer’s Disease (AD). Red light therapy has been indicated as a non‐invasive way of reducing anxiety, mood and sleep optimization in neurodegenerative disorders but its endogenous mechanisms are insufficiently comprehended. Hence, we assessed the effects of scheduled red‐light exposure on clock genes‐*Bmal1* and *Per* 1 expression, feacal boli frequency, and anxiety‐like responses in prolonged LED‐light exposed rats.

**Method:**

Anxiety‐like indices of male rats was assessed using standard neurobehavioural methods under various light settings exposures (n = 5) in groups: I) 12‐hour light/12‐hour dark (NC), II) 12‐hour light and 12‐hour dark + scheduled red light (PC), III) 24‐hour LED light, and IV) 24‐hour LED light + scheduled red light; every two weeks for a total of 60 days. Total RNA was isolated from the rats' basal ganglia and reverse transcription was performed with the ProtoScript cDNA synthesis kit. Quantification and purity of cDNA were assessed using 1μl in a DeNovix spectrometer. Sequenced primers were utilized for the analysis of *m*RNA expression levels of *Bmal*1, *Per*1, and the housekeeping gene (*GAPDH*) through real‐time PCR. The PCR products were resolved by electrophoresis on 1.5% agarose gels and visualized by SafeView staining.

**Result:**

Feacal bolus frequency (p<0.001), closed arm entries (p<0.05), and time spent in the dark area of the light‐dark box (p<0.001) were significantly reduced in the 24‐h LED light+scheduled red light and control groups rats compared to the 24‐h LED light. Real‐time gene expression analyses of *Per*1 showed a significant decline in the 24‐hour LED‐exposed rats, as indicated by decreased *m*RNA expression levels (p<0.05), when compared to other groups. *Bmal*1 gene expression remained unchanged in groups I and II while AGE revealed 100bp and 150 bp banding patterns for *Bmal*1 and *Per*1, respectively.

**Conclusion:**

The findings from this study revealed that red light therapy attenuates LED light‐exposure‐induced anxiogenic responses and *Per*1 gene expression impairment. Consequently, these findings have the potential to inform the development of non‐invasive treatment strategies targeting the improvement of sleep quality in the management of neurodegenerative disorders. However, safety assessments should be ensured for its validation.

